# White matter tract myelin maturation and its association with general psychopathology in adolescence and early adulthood

**DOI:** 10.1002/hbm.24842

**Published:** 2019-10-29

**Authors:** Lucy D. Vanes, Michael Moutoussis, Gabriel Ziegler, Ian M. Goodyer, Peter Fonagy, Peter B. Jones, Edward T. Bullmore, NSPN Consortium, Raymond J. Dolan

**Affiliations:** ^1^ Max Planck UCL Centre for Computational Psychiatry and Ageing Research University College London London UK; ^2^ Wellcome Centre for Human Neuroimaging University College London London UK; ^3^ Institute of Cognitive Neurology and Dementia Research Otto‐von‐Guericke‐University Magdeburg Magdeburg Germany; ^4^ Department of Psychiatry University of Cambridge Clinical School Cambridge UK; ^5^ Department of Clinical, Educational and Health Psychology University College London London UK

**Keywords:** cingulum, general psychopathology, myelin, structural connectivity, uncinate fasciculus

## Abstract

Adolescence is a time period associated with marked brain maturation that coincides with an enhanced risk for onset of psychiatric disorder. White matter tract myelination, a process that continues to unfold throughout adolescence, is reported to be abnormal in several psychiatric disorders. Here, we ask whether psychiatric vulnerability is linked to aberrant developmental myelination trajectories. We assessed a marker of myelin maturation, using magnetisation transfer (MT) imaging, in 10 major white matter tracts. We then investigated its relationship to the expression of a general psychopathology “*p*‐factor” in a longitudinal analysis of 293 healthy participants between the ages of 14 and 24. We observed significant longitudinal MT increase across the full age spectrum in anterior thalamic radiation, hippocampal cingulum, dorsal cingulum and superior longitudinal fasciculus. MT increase in the inferior fronto‐occipital fasciculus, inferior longitudinal fasciculus and uncinate fasciculus was pronounced in younger participants but levelled off during the transition into young adulthood. Crucially, longitudinal MT increase in dorsal cingulum and uncinate fasciculus decelerated as a function of mean *p*‐factor scores over the study period. This suggests that an increased expression of psychopathology is closely linked to lower rates of myelin maturation in selective brain tracts over time. Impaired myelin growth in limbic association fibres may serve as a neural marker for emerging mental illness during the course of adolescence and early adulthood.

## 1 INTRODUCTION

Abnormal neural connectivity due to structural changes in connecting white matter pathways is implicated in a wide range of psychiatric disorders including schizophrenia (Klauser et al., 2017; Kubicki et al., 2007), autism (Karahanoğlu et al., 2018), depression (Tham, San Woon, Sum, Lee, & Sim, 2011), bipolar disorder (Nortje, Stein, Radua, Mataix‐Cols, & Horn, 2013), obsessive–compulsive disorder (Brennan, Rauch, Jensen, & Pope Jr, 2013) and attention‐deficit hyperactivity disorder (Nagel et al., 2011). As a key determinant of white matter integrity, myelin changes, in particular, are thought likely to contribute significantly to a range of psychiatric symptoms. Myelin underpins the efficiency and speed of neuronal signal conduction, enabling rapid communication between disparate brain regions and efficient functional integration during cognitive processing. White matter changes linked to myelin are associated with cognitive function both in health (Lu et al., 2011; Mabbott, Noseworthy, Bouffet, Laughlin, & Rockel, 2006; Nagy, Westerberg, & Klingberg, 2004) and pathology (Vanes et al., 2019; Vanes, Mouchlianitis, Wood, & Shergill, 2018). Individual differences in white matter myelination may thus be a general risk factor for or an outcome of psychiatric disorders (Fields, 2008).

Psychiatric vulnerability has recently been described within the framework of a general psychopathology factor (or “*p*” factor), proposed to reflect a general latent behavioural risk for common psychiatric disorders (Caspi et al., 2014; Lahey et al., 2012). The success of the *p*‐factor in capturing common and shared variance across psychiatric disorders (Krueger & Eaton, 2015) has contributed to a shift of mental health research away from categorical towards more dimensional approaches. The *p*‐factor has been characterised in childhood (Martel et al., 2017) and early adolescence (Carragher et al., 2016; Patalay et al., 2015) through to early adulthood (Laceulle, Vollebergh, & Ormel, 2015; St Clair et al., 2017). It is reported as relatively stable throughout development (Murray, Eisner, & Ribeaud, 2016), capturing familial risk of psychopathology (Martel et al., 2017), and is thought to be subject to genetic regulation (Brikell et al., 2018; Neumann et al., 2016; Selzam, Coleman, Caspi, Moffitt, & Plomin, 2018). Importantly, it is widely accepted that psychiatric symptoms (including those seen in psychosis, depression or anxiety) can occur in the population at large and therefore be manifest patterns of behavioural phenotypes spanning both healthy and clinical populations. Individual variation entails that transdiagnostic and extended phenotypes will be found in community cohorts with shared environmental or familiar features noted in clinical populations (van Os & Reininghaus, 2016). As such, studying behavioural characteristics that exemplify psychopathology in healthy samples can elucidate putative mechanisms of subclinical mental health symptom patterns that are phenomenologically and temporally continuous with actual clinical symptom expression. In healthy individuals, the neural substrate of the *p*‐factor appears to encompass a distributed network of brain structures. Volumetric studies implicate grey matter reductions in prefrontal (Snyder, Hankin, Sandman, Head, & Davis, 2017), striatal (Gong et al., 2019), occipital and cerebellar regions (Moberget et al., 2019; Romer et al., 2018) in association with greater expression of *p*. In terms of white matter integrity, commonly indexed using diffusion imaging metrics, reduced integrity in callosal (Riem et al., 2019) and pontine‐cerebellar (Romer et al., 2018) pathways are also linked to the expression of a *p*‐factor. However, positive associations with corpus callosum integrity have also been observed (Hinton et al., 2019), hinting that neurodiversity rather than a simple deficit accompanies variation in *p*. Varied findings are also reported in studies of resting‐state functional connectivity, with a general psychopathology factor relating to reduced fronto‐temporal connectivity (Alnæs et al., 2018) as well as delayed maturation of the default mode network (DMN) (Sato et al., 2016), but also with increased connectivity of visual cortex with the DMN and frontoparietal networks (Elliott, Romer, Knodt, & Hariri, 2018). The considerable heterogeneity of these findings, likely reflecting methodological differences, suggests that multiple core networks may be involved in conferring diversity and associated vulnerability. Structural connectivity within and between these networks, contingent on healthy development of white matter tracts, is of particular interest in this respect.

A significant shortcoming of most neuroimaging studies is the lack of a longitudinal design needed to fully address the relationship between brain maturation and *p*‐factor variation, particularly with a focus on developmental myelin‐sensitive measures. Myelination unfolds throughout adolescence and into early adulthood, coinciding with the very period of enhanced risk for a range of psychiatric disorders. This conjunction warrants investigation of a link between myelin maturation and differences in *p*‐factor scores indexing individual variation in psychiatric risk (Baumann & Pham‐Dinh, 2001; Tau & Peterson, 2010). Longitudinal studies offer unique advantages over cross‐sectional designs, particularly greater consistency and sensitivity to the rate of change during development (Barrick, Charlton, Clark, & Markus, 2010). In addition, longitudinal designs allow for an investigation of ongoing changes within specific developmental periods at an individual level (as opposed to differences between subjects or cohorts). Studying brain regional rates of myelin development is particularly important as it avoids inferring maturational status from mean myelin levels, but instead focusses on how the trajectory of growth is unfolding (Simmonds, Hallquist, Asato, & Luna, 2014). For example, we recently showed in otherwise healthy adolescents that a reduction in an expected longitudinal increase of a cortical myelin marker is associated with domain‐specific traits of compulsivity and impulsivity (Ziegler et al., 2019). Aberrant myelin maturation has also been linked to clinical diagnosis, for example, adolescent‐onset schizophrenia (Douaud et al., 2009). Therefore, leveraging longitudinal designs to study the rate of change in white matter myelin with respect to a general liability for mental illnesses can offer novel insights into transdiagnostic connectivity markers of mental disorders.

We used an accelerated longitudinal design to study myelin maturation in the major white matter tracts of adolescents and young adults using magnetisation transfer (MT) saturation imaging. This marker shows high sensitivity to myelin and related macromolecules (Schmierer, Scaravilli, Altmann, Barker, & Miller, 2004; Turati et al., 2015; Weiskopf, Mohammadi, Lutti, & Callaghan, 2015), bestowing it with greater specificity over complementary markers, such as diffusion metrics. Intracortical and juxtacortical myelin maturation has previously been described in this cohort (Romero‐Garcia et al., 2018; Whitaker et al., 2016; Ziegler, Hauser, et al., 2019). Here, we focus on tract‐specific MT in order to characterise if longer‐range structural connectivity changes confer explicit risk to reported mental health problems.

We examined all tracts included within an openly available tractography atlas (Hua et al., 2008), based on the consideration that these have been shown to be sufficiently reproducible to be used as a tool for quantitative analyses (Wakana et al., 2007). These include association tracts (inferior fronto‐occipital fasciculus [IFOF], inferior longitudinal fasciculus (ILF), superior longitudinal fasciculus (SLF), uncinate fasciculus (UF), dorsal and hippocampal cingulum), projection tracts (anterior thalamic radiation [ATR], corticospinal tract [CST]), as well as anterior and posterior aspects of the commissural corpus callosum (forceps minor and forceps major). To assess individual differences in *p*‐factor scores we followed St Clair et al. (2017), estimating a latent general *p*‐factor (also termed “distress”) from a comprehensive set of self‐report questionnaires. We modelled longitudinal and cross‐sectional age effects on the distribution of myelin in pre‐defined tracts, as well as the relationship between the *p*‐factor and myelin maturation in these target regions. Based on previous research on white matter development (Lebel & Beaulieu, 2011; Lebel, Treit, & Beaulieu, 2017; Simmonds et al., 2014), we expected to see a trajectory of myelin maturational increase in the majority of target tracts throughout adolescence and a reduced myelin growth in subjects experiencing higher levels of reported distress.

## 2 MATERIALS AND METHODS

### 2.1 Sample

A total of 318 healthy adolescents and young adults between the ages of 14 and 24, recruited in London and Cambridgeshire, underwent MRI scanning as part of the *Neuroscience in Psychiatry Network project. Subjects were selectively recruited from a larger questionnaire cohort (*N* > 2,400) to ensure sex, age and ethnicity distribution representative of the community. Exclusion criteria were any self‐reported neurological, developmental or psychiatric disorders. Subjects were scanned at baseline and a subset returned for a follow‐up scan on average 1.3 (*SD* = 0.3) years later. After quality control, data from 293 subjects were included in further analyses. The study was approved by the Cambridge Central Research Ethics Committee (12/EE/0250) and all participants (and their legal guardian where participants were under 16 years old) gave written informed consent.

### 2.2 P‐factor

We utilised *p*‐factor or “general symptomatology” scores at baseline and follow‐up, as previously validated and published by St Clair et al. (2017), where full modelling details and fit indices can be found. In brief, exploratory and confirmatory factor analyses yielded an optimal model with a bifactor structure, including a general *p*‐factor and five specific factors. The general factor explained 92% of the reliable variance (Rodriguez, Reise, & Haviland, 2016) in total scores and as such, the specific factors explained little beyond *p*. Due to this and our a priori interest in general psychopathology, the specific factors were not the focus of further analysis here. Analyses were conducted at item level on items derived from self‐report measures of anxiety (Revised Children's Manifest Anxiety Scale), depression (Moods and Feelings Questionnaire), psychotic‐spectrum symptoms (Schizotypal Personality Questionnaire), obsessionality (Revised Leyton Obsessional Inventory), antisocial behaviour (Antisocial Behaviour Questionnaire, self‐esteem (Rosenberg Self‐Esteem Questionnaire) and well‐being (Warwick‐Edinburgh Mental Well‐Being Scale) (Goodyer et al., 2011; Kiddle et al., 2017). Here, we assess *p‐*factor scores in the MRI subsample; however, importantly, bifactor analysis was conducted on the full questionnaire cohort (*N* = 2,228) (St Clair et al., 2017), resulting in more reliable and representative factor scores. As a result, the MRI sample demonstrated satisfactory stability of scores over time, with a correlation of *R* = .65 (*p* < .001) between baseline and follow‐up *p*‐factor scores.

### 2.3 MRI acquisition and processing

Subjects were scanned on identical Siemens Magnetom TIM Trio whole‐body 3T MRI scanners in Cambridge and London as per the multi‐parameter mapping (MPM) protocol, which has previously undergone multi‐centre validation and is described in detail elsewhere (Weiskopf et al., 2013; Weiskopf et al., 2015). Acquisition parameters were identical across sites. Whole‐brain multi‐echo FLASH MT weighted contrast were acquired at 1 mm isotropic resolution (TR: 23.7, *α* = 6°, 176 sagittal slices, FOV = 256 × 240 mm^2^, matrix = 256 × 240 × 176). Semi‐quantitative MT saturation maps were derived using biophysical models as described in more detail in Helms, Dathe, Kallenberg, and Dechent (2008) and Tabelow et al. (2019) using the hMRI toolbox (http://www.hmri.info) for SPM (Wellcome Centre for Human Neuroimaging, London, UK, http://www.fil.ion.ucl.ac.uk/spm). These MT maps represent the percentage of signal loss as a result of an off‐resonance MT pre‐pulse, which preferentially saturates the macro‐molecular bound water pool, known to be a sensitive measure for myelin content (Schmierer et al., 2004; Turati et al., 2015). In contrast to the commonly used MT ratio, this semi‐quantitative MT saturation metric explicitly removes bias induced by spatially varying T1 relaxation times and flip angle inhomogeneities.

MT maps were longitudinally pre‐processed using a custom pipeline, as described in detail in Ziegler, Hauser, et al. (2019). Briefly, each subject's baseline and follow‐up scans were first registered to each other using symmetric diffeomorphic registration (Ashburner & Ridgway, 2013), resulting in a midpoint image for each subject. Midpoint images were subsequently segmented using the Computational Anatomy Toolbox (CAT, http://www.neuro.uni-jena.de/cat/). The maps were then normalised to MNI space using SPM's geodesic shooting and tissue‐weighted smoothing with a Gaussian kernel of 3 mm full width at half maximum was applied. Tissue‐weighted smoothing (Draganski et al., 2011) was chosen so as to preserve MT values within grey/white matter tissue classes and thereby account for small discrepancies in spatial normalisation, ensuring that obtained MT values within the tracts would not be contaminated by non‐white matter tissue values.

Rigorous quality assessment included manual inspection for motion artefacts by an expert (G.Z.) and the use of statistical covariance‐based inhomogeneity measures (as implemented in the CAT toolbox), which detects subjects with extreme overall deviation of quantitative values. In addition, as a proxy of motion during the scan, the *SD* parameter of R2* exponential decay residuals (SDR2*) in white matter areas was computed. This has been shown to provide a reliable measure of motion across scans in the context of MPMs (Castella et al., 2018; Ziegler, Hauser, et al., 2019). Scans with SDR2* values above 2.5 *SD*s from the sample mean were removed from all further analyses.

The final data set included in our analyses consisted of 283 baseline scans and 203 follow‐up scans (from a total of 293 subjects).

### 2.4 Region of interest histogram extraction

We defined 10 tract ROIs based on the JHU White Matter Tractography Atlas (Hua et al., 2008) corresponding to the ATR, dorsal cingulum bundle, hippocampal cingulum bundle, corticospinal tract (CST), forceps major, forceps minor, inferior fronto‐occipital fasciculus (IFOF), ILF, SLF and UF. Probability maps for each of these tracts were thresholded at 20% to minimise partial voluming. Separate unilateral masks for each hemisphere were created for all ROIs except for forceps major and forceps minor, which remained bilateral. For each MT map, we summed the number of voxels within each ROI for 100 uniform bins within a range of MT values from 0.8 to 2.2. We then normalised each histogram with respect to its area and extracted the histogram mean (first moment), variance (second moment) and skewness (third moment). Therefore, each ROI yielded three metrics for every subject at each available time point within each hemisphere (with the exception of forceps major and minor, which were not split by hemisphere).

### 2.5 Statistical analysis

#### 2.5.1 Modelling tract‐wise MT changes

ROI histogram mean, as an index of average level of myelination within a tract, was the primary dependent variable in the following analyses. We performed model comparisons for each ROI in order to identify tracts showing significant developmental MT increase in our cohort. First, to disentangle longitudinal from cross‐sectional age effects, we deconstructed *age* into separate within‐subject (longitudinal) and between‐subject (cross‐sectional) components (as recommended in Neuhaus & Kalbfleisch, 1998). Specifically, cross‐sectional age for a subject *i* was calculated as ${\bar{\mathrm{age}}}_{i}-\bar{\mathrm{age}}$ (where ${\bar{\mathrm{age}}}_{i}\;$ is subject *i*'s mean age across visits and $\bar{\mathrm{age}}$ is the mean age across the whole sample), thus representing a subject's mean centred age with respect to the sample. Longitudinal age for subject *i* at time point *j* was calculated as $\mathrm{ag}e_{\mathrm{ij}}-{\bar{\mathrm{age}}}_{i}$, thus representing the within‐subject centred deviation from the subject's own mean age. This distinction allows an assessment of true within‐subject change (taking into account the temporal distance between two scans) independent of cross‐sectional age effects.

For each ROI, histogram means were modelled using linear mixed‐effects models (Pinheiro, Bates, DebRoy, Sarkar, & R Development Core Team, 2013), allowing for a random intercept for each subject. Covariates included in all models were sex, ethnicity, total intracranial volume, subject motion, acquisition site, and, where applicable, tract laterality (left vs. right). A null model (M0) including these covariates alone was fit as a first step. Next, a model (M1) was fit including these covariates as well as the main effects of longitudinal and cross‐sectional age. Finally, an additional interaction between longitudinal and cross‐sectional age was added (M2), which assesses whether longitudinal change in MT differs across the sample's age range. Model comparisons using likelihood ratio F tests were conducted to identify the winning model (M0, M1 or M2) for each ROI. Lastly, we tested individually whether the winning model could be improved with the additions of sex × longitudinal age, sex × cross‐sectional age, laterality × longitudinal age or laterality × cross‐sectional age interaction terms, thereby assessing whether age effects differed for the two hemispheres or the two sexes. All model comparisons were Bonferroni corrected for the set of 10 regions, resulting in a significance threshold of *p* < .05/10 = .005 used throughout.

#### 2.5.2 Assessing associations between general psychopathology and MT changes

Next, we tested for associative effects of *p*‐factor scores on changes in MT within tracts identified in the previous step as showing a significant longitudinal increase in our sample. To achieve this, we deconstructed the *p‐*factor into longitudinal and cross‐sectional components in an identical fashion to *age*. Thus, cross‐sectional *p*‐factor scores represent a subject's average level of *p* across both visits, relative to the whole sample average. Longitudinal *p*‐factor scores represent the (within‐subject centred) level of *p* for each time point. This approach enables us to disentangle the effects of *overall* differences in *p*‐factor scores on MT from the effects of *change* in *p* on MT.

We compared the winning model from the previous step to an identical model including additional main effects of longitudinal *p*‐factor, cross‐sectional *p*‐factor, as well as an interaction between cross‐sectional *p*‐factor and longitudinal age (assessing whether longitudinal MT change differs across levels of general psychopathology). If the previously winning model included a significant interaction term between longitudinal and cross‐sectional age (i.e., M2), this was also compared to an additional model including the three‐way interaction with cross‐sectional *p*‐factor (and lower order two‐way interactions). Finally, we tested whether the winning model would be improved with the addition of an interaction between sex and the highest order term involving *p*‐factor (e.g., sex × cross‐sectional *p*‐factor × longitudinal age).

Although our main hypothesis pertained to an association between *p*‐factor scores and MT in tracts still undergoing maturation, for completeness we also assessed effects of *p* on MT in tracts that did not show any longitudinal MT change overall. This was to ensure that we would not miss overall growth effects due to large amounts of variance induced by inter‐individual differences in psychopathology. For this reason, a significance threshold of *p* < .005, correcting for all ROIs, was retained for these analyses.

#### 2.5.3 Developmental effects on histogram shape

To fully characterise the potential effects of age on the distribution of MT values in each tract, we conducted identical model comparisons for histogram variance and skewness. These exploratory analyses reflect the possibility that myelin maturation is not only characterised by a mean increase in myelin but also, for example, increased tissue homogeneity (i.e., narrowing of the distribution of MT values) and/or a change in the symmetry of MT distributions within tracts.

## 3 RESULTS

Mean histograms for each ROI (normalised with respect to the area for illustrative purposes) at baseline by laterality and cross‐sectional age bin are presented in Supplementary Figure 1.

### 3.1 Identifying tracts showing developmental increase of myelin‐sensitive MT

The null model (M0) was not significantly improved by the addition of age in the CST (*χ* ^*2*^ = 1.31, *p* = .520), forceps major (*χ* ^*2*^ = 2.64, *p* = .267) and forceps minor (*χ* ^*2*^ = 5.20, *p* = .074), suggesting there were no (longitudinal or cross‐sectional) age effects on mean MT in these tracts.

**Figure 1:**
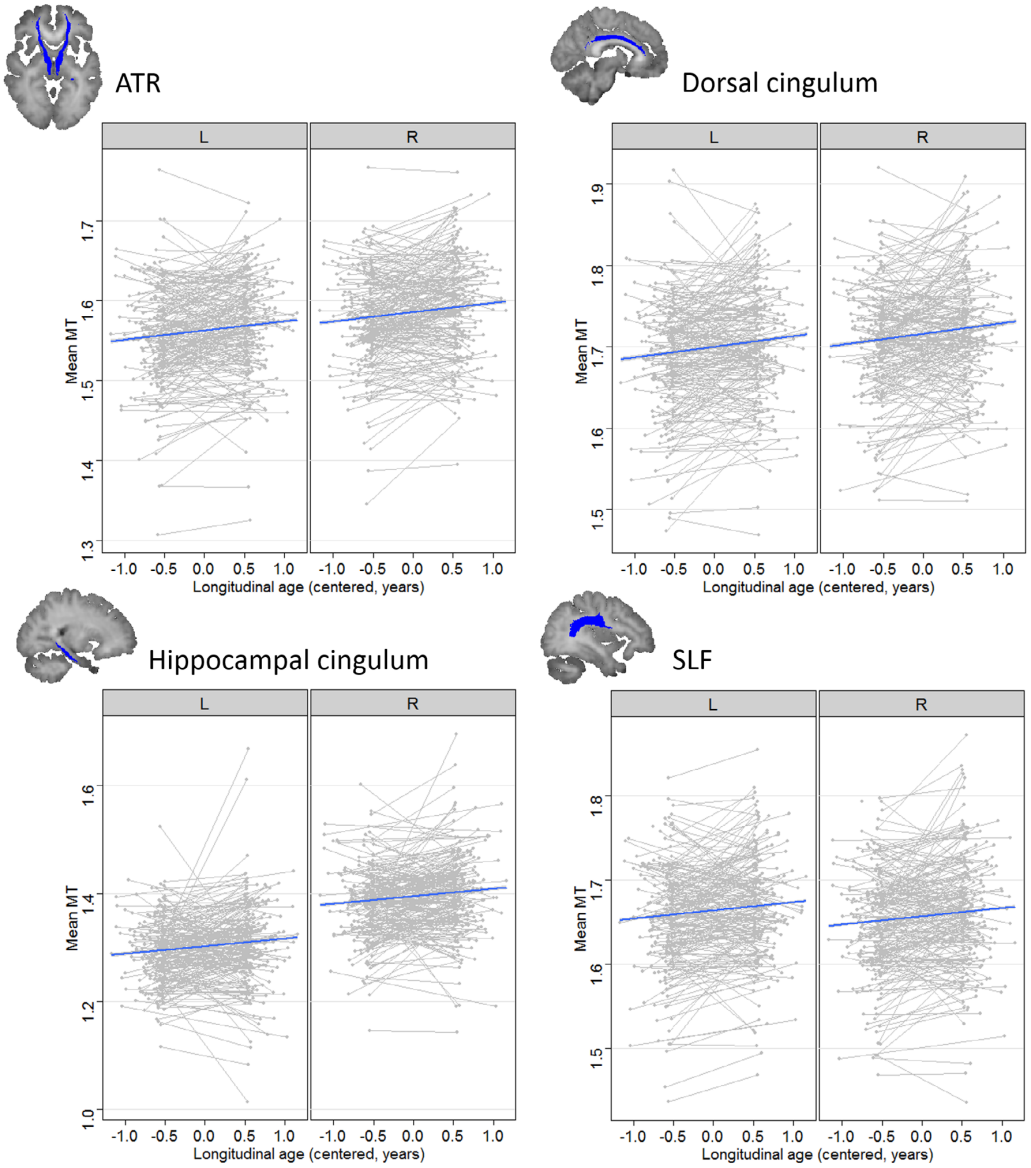
Longitudinal change in mean magnetisation transfer (MT) in the anterior thalamic radiation (ATR), dorsal cingulum, hippocampal cingulum and superior longitudinal fasciculus (SLF), by laterality. MT significantly increases with a longitudinal age

The additive model (M1) including main effects of longitudinal and cross‐sectional age was the winning model for the ATR (*χ* ^*2*^ = 36.34, *p* < .0001), dorsal cingulum (*χ* ^*2*^ = 32.63, *p* < .0001), hippocampal cingulum (*χ* ^*2*^ = 37.83, *p* < .0001) and SLF (*χ* ^*2*^ = 23.25, *p* < .0001). All four ROIs showed a significant positive effect of longitudinal age (all *p*s < .0001), indicating a within‐subject increase of MT in these tracts (see Figure 1). Only the hippocampal cingulum showed an additional significant effect of cross‐sectional age, indicating higher MT in older subjects in this cohort, *p* = .002. A significant effect of laterality indicated higher MT values in the right compared to the left hemisphere for the ATR, dorsal cingulum, hippocampal cingulum, IFOF and ILF, and higher MT in the left compared to right hemisphere for the SLF and UF, all *p* < .001.

**Figure 2:**
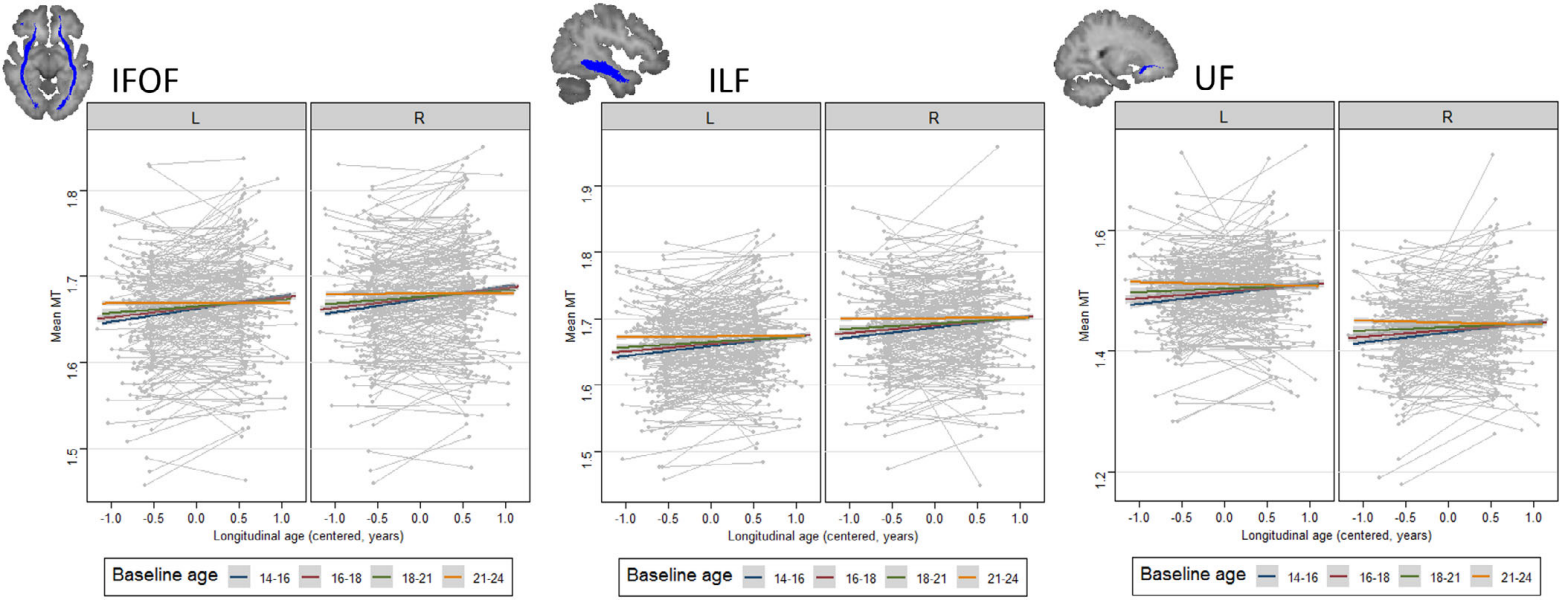
Longitudinal change in mean magnetisation transfer (MT) as a function of cross‐sectional age (baseline age is used for illustrative purposes) in the inferior fronto‐occipital fasciculus (IFOF), inferior longitudinal fasciculus (ILF) and uncinate fasciculus (UF), by laterality. MT increase is strongest in the youngest participants and levels off in older participants

There was a significant negative interaction between longitudinal age and cross‐sectional age (M2) in the IFOF (*χ* ^*2*^ = 11.40, *p* < .0001), ILF (*χ* ^*2*^ = 9.39, *p* = .002) and UF (*χ* ^*2*^ = 8.66, *p* = .003). As seen in Figure 2, longitudinal MT increase was strongest in youngest adolescents in these tracts, whereas virtually no increase was seen in early adulthood. IFOF and ILF showed higher MT values in the right compared to the left hemisphere, whereas there were higher mean MT values in the left compared to the right UF, all *p*s < .001. Neither the rate of change of MT nor cross‐sectional age effects on MT differed significantly between the two hemispheres or between the two sexes in any of the reported ROIs.

### 3.2 Association between development of MT and *p*‐factor scores

**Figure 3:**
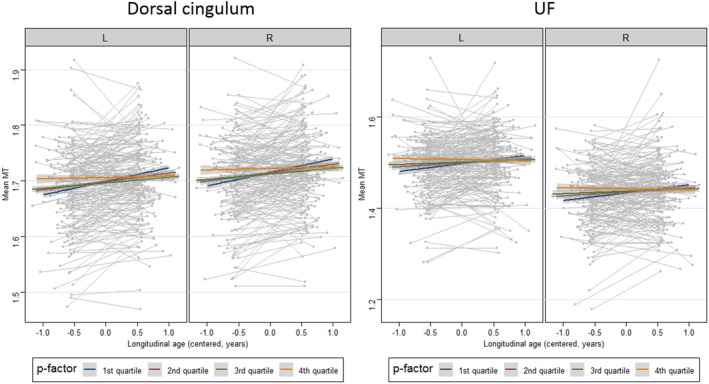
Longitudinal change in mean magnetisation transfer (MT) as a function of *p*‐factor (distress) in the dorsal cingulum and uncinate fasciculus (UF), by laterality. MT increase is strongest in participants with low *p‐*factor scores and flattens out with increasing psychopathology

We conducted additional model comparisons for tracts showing significant MT increase (ATR, dorsal and hippocampal cingulum, SLF, IFOF, ILF and UF) as well as tracts not showing an overall MT increase (CST, forceps major and forceps minor). Including the *p‐*factor in the model improved the model significantly for dorsal cingulum (*χ* ^*2*^ = 20.56, *p* < .0001) and UF (*χ* ^*2*^ = 13.13, *p* = .004). In both of these tracts, there was a significant negative interaction between cross‐sectional *p*‐factor and longitudinal age, indicating that longitudinal MT change over study visits in the dorsal cingulum and UF differed as a function of *p*. As seen in Figure 3, subjects showing high *p*‐factor scores showed the least MT increase, whereas subjects with low *p*‐factor scores showed the strongest MT increase in these tracts. The models for both tracts were not improved by the addition of a three‐way interaction between cross‐sectional *p*‐factor, longitudinal age and sex, indicating that the depicted effect did not differ between male and female participants.

None of the models revealed significant main effects of cross‐sectional or longitudinal *p*‐factor, indicating no direct correlation between mean levels of *p* and mean level of myelination, and no correlation between longitudinal change in *p* and longitudinal change in MT.

### 3.3 Histogram variance and skewness

Histogram variance in the CST and forceps minor, as well as skewness in the CST and ATR, were best explained by a model including longitudinal and cross‐sectional age effects (all *χ* ^*2*^ > 16.08, *p* < .005), as seen in Supplementary Table 1. Specifically, histogram variance in the CST and forceps minor decreased as a function of cross‐sectional age, indicating a narrower distribution of MT values in older subjects in these regions. Skewness in the ATR and CST was also negatively impacted by cross‐sectional age, suggesting that older subjects exhibit more negatively skewed distributions compared to younger subjects. In the CST, this tendency was also reflected in a negative effect of longitudinal age on histogram skewness, *p* < .001. There were no significant age by laterality interactions in any of these tracts. Furthermore, the addition of the *p*‐factor did not improve model fit for any of the ROIs. Further details of ROI‐specific model comparisons for histogram variance and skewness are found in Supplementary Table 1.

## 4 DISCUSSION

We investigated white matter development using MT imaging in 10 pre‐defined white matter tracts in healthy participants aged 14–24, assessing the relationship of myelin‐sensitive MT to latent psychopathology (“*p*”‐factor) scores, thought to index general psychiatric vulnerability. Several tracts, including bilateral ATR, cingulum and SLF showed an MT increase through adolescence into early adulthood. Other tracts including IFOF, ILF and UF showed longitudinal increases that were evident in younger participants but slowed with cross‐sectional age. In addition, availing of multiple distributional descriptors in each tract, we found that age‐related MT changes in some projection and callosal fibres were best captured by change in variance and skewness, over and above the mean, potentially reflecting distinct developmental microstructural processes within these regions. In terms of associations with psychopathology, MT increase in the dorsal cingulum and UF decelerated with higher *p*‐factor scores, indicating that myelin maturation in these frontal tracts may be slowed in individuals with higher levels of psychiatric symptoms.

### 4.1 Developmental findings

MT, likely reflecting myelin (Turati et al., 2015), increased in the majority of white matter tracts throughout adolescence, a finding that aligns with much existing literature on white matter development, albeit a literature that mostly relies on diffusion imaging metrics (Bava et al., 2010; Krogsrud et al., 2016; Lebel, Walker, Leemans, Phillips, & Beaulieu, 2008; Schmithorst & Yuan, 2010; Simmonds et al., 2014; Wang et al., 2012). These metrics, such as fractional anisotropy (FA), tend to be sensitive to white matter microstructural change, but are less specific to myelin changes per se (Beaulieu, 2002; Wheeler & Voineskos, 2014). Recent reviews of developmental change using diffusion metrics indicate a consensus in a view that there are considerable regional differences in both rate and timing of white matter maturation (Lebel et al., 2017; Lebel & Deoni, 2018; Schmithorst & Yuan, 2010; Tamnes, Roalf, Goddings, & Lebel, 2018). Our study lends support to a literature, which suggests a protracted maturation of frontotemporal connections, including within the cingulum and SLF that extends well into adulthood (Lebel et al., 2008; Tamnes et al., 2009). Maturation of dorsal cingulum, in particular, has been shown to occur much later compared to other tracts (Lebel et al., 2017; Simmonds et al., 2014). However, we did not replicate a similar pattern of late maturation of the UF, which is often observed in terms of FA (Lebel et al., 2017). Instead, in our data, the longitudinal change in UF decelerated post‐adolescence. This observation aligns more closely with findings of earlier UF maturation in terms of mean diffusivity (MD) compared to FA (Lebel et al., 2008; Wang et al., 2012), indicating that MD may be more reflective of myelin changes.

We found evidence for a deceleration in myelin change in the IFOF and ILF, manifest as MT increase levelling off with age. This is consistent with reports that both these tracts reach 90% of their adult FA values before adulthood, and certainly earlier than the cingulum bundle, which continues to undergo change up to the late 20s (Lebel et al., 2008; Simmonds et al., 2014). Nonlinear trajectories of white matter development have been reported in a number of large neuroimaging studies, with regionally specific peaks reached at variable time points in late adolescence and early adulthood (Lebel & Deoni, 2018). We note that nonlinear development could not be explicitly modelled longitudinally in our analysis due to the presence of only two scan time points (Fjell et al., 2010), though we were able to test for nonlinear development indirectly by assessing whether longitudinal change differed over the sampled age range.

We found no evidence for age‐dependent myelin growth in the forceps major and minor, compatible with previous diffusion imaging findings that microstructural development in the anterior (genu) and posterior (splenium) corpus callosum is mostly complete by early adolescence (Lebel et al., 2008; Pohl et al., 2016; Rollins et al., 2010). Callosal microstructural changes in adolescence and adulthood, if present, are more frequently reported in the callosum body (Lebel & Beaulieu, 2011; Rollins et al., 2010), which was not included in our analyses. The forceps major, containing the splenium of the corpus callosum, shows a particularly fast FA increase before the age of 13 (Mah, Geeraert, & Lebel, 2017), consistent with a posterior‐to‐anterior gradient of myelin maturation in the brain.

Interestingly, we did not observe any longitudinal change in mean MT within the CST. This is inconsistent with previous studies suggesting that CST is one of the latest maturing tracts in terms of diffusion parameters (Lebel et al., 2017). The absence of MT increase in our sample could be due to the specific studied age range, as slopes may temporarily flatten off consistent with the idea of a putative interim period in some tracts during which there is no myelin growth during adolescence (Simmonds et al., 2014). However, intriguingly, we observed an effect of cross‐sectional age on both variance and skewness of MT in the CST, findings reflecting a narrowing of the MT distribution with age as well as a shift of the mass of the distribution towards higher values. The latter was also evident longitudinally. MT saturation imaging and diffusion parameters likely index different aspects of white matter microstructure, with MT showing higher specificity for myelin. It has been suggested that discrepancies in the developmental findings from different white matter neuroimaging techniques might be explained by a greater sensitivity of diffusion metrics to, for example, axonal packing (Lebel & Deoni, 2018). As such, a more protracted change in axonal packing (rather than or in addition to myelin per se) might be more readily reflected in a change in variance (rather than the mean) of MT values due to increased tissue homogeneity. Furthermore, if myelin maturation does indeed continue for longer on average than is the case for other tracts, but shows greater variability in the exact timing of the peak, this may be more readily reflected in changes in skewness than in mean values. This highlights the importance of not only cross‐modal imaging and replication but also the use of within‐modality metrics that go beyond simple averages (which also appear to be differentially sensitive to longitudinal and cross‐sectional effects).

### 4.2 P‐factor findings

Crucially, we found that increased expression of *p*‐factor scores was associated with reduced rates of MT increase, which we interpret as slower myelination, in dorsal cingulum and UF. Both tracts constitute core‐connecting pathways of distinct limbic networks and have been implicated in several neuropsychiatric disorders (Buckholtz & Meyer‐Lindenberg, 2012; Passamonti et al., 2012). The temporo‐amygdala‐orbitofrontal network, connected through UF, is implicated in the integration of emotion and cognition (Catani, Dell'Acqua, De Schotten, & Reviews, 2013), and is particularly important for processes such as flexible reward learning (Schoenbaum, Roesch, Stalnaker, & Takahashi, 2009; Stalnaker, Franz, Singh, & Schoenbaum, 2007) and emotional memory (LaBar & Cabeza, 2006; Rauch et al., 1996). The DMN, the medial portion of which (posterior cingulate/precuneus to anterior cingulate/medial prefrontal cortex) is connected through the dorsal cingulum, shows deactivation during goal‐directed tasks and is implicated in introspective and self‐referential thought (Raichle et al., 2001).

Functional and structural imaging studies point towards an involvement of the DMN and temporo‐amygdala‐orbitofrontal networks in general psychopathology during development. In one study, delayed maturation of functional connectivity within the DMN was associated with childhood psychopathology (Sato et al., 2016), while in young adults functional hyperconnectivity between visual association cortex and DMN has been associated with higher *p*‐factor scores (Elliott et al., 2018). A recent cross‐sectional diffusion imaging study in over 700 healthy adolescents showed that general psychopathology, as well as cognitive performance, was associated with frontotemporal white matter disconnectivity at the intersection of the UF and IFOF (Alnæs et al., 2018). Consistent with our findings, the latter suggests that the structural integrity of frontotemporal circuits may be a transdiagnostic brain phenotype for psychiatric vulnerability. Our results extend these earlier findings in two crucial ways: first, we show that myelination may underpin this association; second, by using a longitudinal design we demonstrate that—with respect to MT—it is the rate of change that is the most relevant feature for general psychopathology during development. Indeed, absolute MT values showed no relationship to *p*‐factor scores in our study, underscoring intra‐ rather than inter‐individual maturational status as a critical factor. Importantly, our findings provide a potential neural mechanism underlying the ability of the *p*‐factor to predict future functioning, as found in adolescents (Patalay et al., 2015) and adults (Lahey et al., 2012) over periods as long as 3 years. In this framework, the persistence of problems likely reflected in the *p*‐factor appears to be underpinned by slowed maturation over time of white matter tracts central to cognitive and emotional processing.

With respect to cortical maturation, our findings dovetail with observations in this same data set that highlight adolescence as a key period for consolidation of highly connected hubs in association cortices, driven in large part by myelination and linked to the expression of genes enriched for risk of schizophrenia (Whitaker et al., 2016). In addition, aberrant myelin development in intra‐ and juxta‐cortical regions has been specifically linked to an expression of compulsive and impulsive traits (Ziegler, Hauser, et al., 2019), highlighting the link between non‐normative longitudinal brain maturation trajectories and psychiatric vulnerability in adolescence.

### 4.3 Limitations and outlook

An open question remains as to whether altered myelin maturation is an underlying cause of increased psychopathology, an adverse outcome thereof, or a concomitant effect of other disrupted brain processes more central to psychopathology. Myelin is known to be subject to substantial experience‐dependent changes (Cicchetti, 2002; Grossman et al., 2003), a notion firmly embedded in the childhood adversity literature (Daniels et al., 2013; Ziegler et al., 2019). For example, chronic hyperactivity of the hypothalamic–pituitary–adrenal axis as a result of life stressors is thought to result in a slowing down of myelin development (Sapolsky, 1992). Conversely, there is tentative evidence that genetic dysregulation of myelin development causally underlies specific psychiatric symptoms, particularly in schizophrenia (Hakak et al., 2001; Nave & Ehrenreich, 2014), a finding strengthened by animal studies that experimentally manipulate genes specifically in oligodendrocytes (Roy et al., 2007). Therefore, the most likely account of the association between neurodevelopment and psychopathology may be one of bidirectional and mutually exacerbating influences between the two (Nigg, 2016), but further neuroepidemiological studies are needed to explicitly address this hypothesis.

A further potential limitation concerns the specificity of MT saturation imaging to myelin content. MT saturation is one of several MRI metrics commonly used to index myelin, including estimations of the myelin water fraction (MWF) (e.g., derived from multicomponent T1 and T2 relaxometry, Deoni, Rutt, Arun, Pierpaoli, & Jones, 2008), T1w/T2w ratio, or the longitudinal relaxation rate R1. While there is evidence for substantial correlations with histological measures of myelin for MT (Schmierer et al., 2004), MWF (Laule et al., 2006) and R1 (Stueber et al., 2014), imperfect correlations between these metrics suggest that each may be differentially sensitive to distinct aspects of tissue microstructure (Geeraert et al., 2017; Hagiwara et al., 2018; O'Muircheartaigh et al., 2019). Quantitative, or semi‐quantitative, MT related metrics are suggested as more robust measures of myelin compared to MWF (Dula, Gochberg, Valentine, Valentine, & Does, 2010) and T1w/T2w ratio (Hagiwara et al., 2018), as well as more specific to myelin compared to R1 and R2* (Callaghan et al., 2014), which show additional sensitivity to iron content. However, MT measures may also be sensitive to the effects of neuroinflammation or oedema (Gareau, Rutt, Karlik, & Mitchell, 2000; Stanisz, Webb, Munro, Pun, & Midha, 2004) and as such remain an imperfect measure of myelin. Future research might usefully address the specificity of our longitudinal findings to myelination by comparing MT saturation effects with other white matter microstructure metrics, an approach adopted in recent work in studies of late childhood and early adolescence, albeit reports on cross‐sectional and not longitudinal data (Geeraert, Lebel, & Lebel, 2019; Moura et al., 2016).

Since we excluded subjects with self‐reported mental health diagnoses, our sample reflects a relatively healthy population at the time of study. This selection approach is in line with the general notion surrounding the *p*‐factor and comes with a number of advantages. First, the *p*‐factor as measured in healthy community cohorts is thought to reflect “distress” that is phenomenologically continuous with clinical expression of psychopathology (Caspi et al., 2014; St Clair et al., 2017) and subject to identical neurodevelopmental, as well as environmental and genetic, influences. Second, several studies in non‐clinical community samples have shown that the *p*‐factor is predictive of later adverse mental health and life outcomes (Laceulle, Chung, Vollebergh, & Ormel, 2019; Lahey et al., 2012; Pettersson, Lahey, Larsson, & Lichtenstein, 2018), suggesting that assessing *p* in healthy individuals, particularly during apparently healthy development, is a useful tool for estimating risk for distal clinical outcomes (Snyder, Young, & Hankin, 2017). Nevertheless, we recognise that certain neurobiological discontinuities may also exist between healthy, subclinical and clinical populations and that future research is necessary to test explicitly the correspondence of neural mechanisms underlying general psychopathology construct in healthy and clinical samples.

## 5 CONCLUSIONS

In summary, we replicate and extend previous findings of protracted white matter development throughout adolescence and early adulthood across the majority of large‐scale white matter tracts, developmental effects most likely reflecting myelin maturation. Furthermore, our results suggest that the rate of myelination in limbic association fibres may be a useful transdiagnostic marker for psychiatric vulnerability during development. The longitudinal design of our study lends weight to this finding. Avenues for future research include investigations into how this association might change across the lifespan including whether it might give rise to distinct clinical phenotypes.

## CONFLICT OF INTEREST

The authors declare no potential conflict of interest.

## Supporting information


**Supplementary Table 1** *Likelihood ratio model comparison results for histogram variance and skewness for each region of interest*. *M0: simple covariate model without age*. *M1: model including additive effects of longitudinal and cross‐sectional age*. *M2: model including additive and interactive effects of longitudinal and cross‐sectional age*. *A corrected significance threshold of p =*. *005 was used*.
**Supplementary Table 2**. *Neuroscience in Psychiatry Network (NSPN) Consortium author list*

**Supplementary Figure 1** Supplementary information

## Data Availability

The data that support the findings of this study are available from the corresponding author upon reasonable request.
