# Supplementary figures and images for: White matter tract myelin maturation and its association with general psychopathology in adolescence and early adulthood
Source: Hum Brain Mapp. 2019 Oct 29;41(3):827–39. doi: 10.1002/hbm.24842 (PMC7268015; doi:10.1002/hbm.24842)

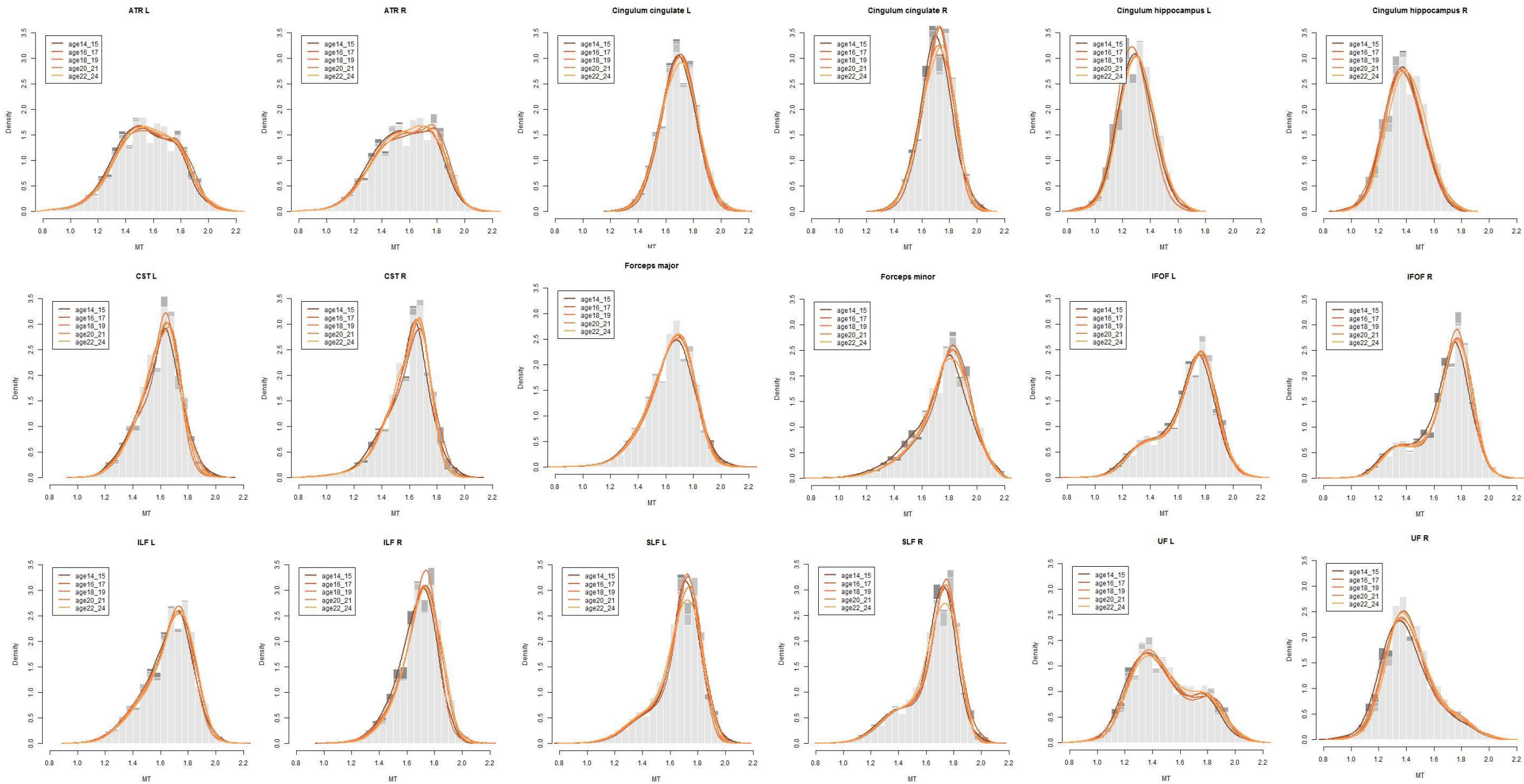

Supplement: Supplementary file 1 — Supplementary Table 1 Likelihood ratio model comparison results for histogram variance and skewness for each region of interest. M0: simple covariate model without age. M1: model including additive effects of longitudinal and cross‐sectional age. M2: model including additive and interactive effects of longitudinal and cross‐sectional age. A corrected significance threshold of p =. 005 was used. Supplementary Table 2. Neuroscience in Psychiatry Network (NSPN) Consortium author list [file HBM-41-827-s001.pdf]
