# Supplementary material for: White matter tract myelin maturation and its association with general psychopathology in adolescence and early adulthood
Source: Hum Brain Mapp. 2019 Oct 29;41(3):827–39. doi: 10.1002/hbm.24842 (PMC7268015; doi:10.1002/hbm.24842)
Supplement: Supplementary file 2 — Supplementary Figure 1 Supplementary information [file HBM-41-827-s002.docx]

**Supplementary Table 1**

*Likelihood ratio model comparison results for histogram variance and skewness for each region of interest. M0: simple covariate model without age. M1: model including additive effects of longitudinal and cross-sectional age. M2: model including additive and interactive effects of longitudinal and cross-sectional age. A corrected significance threshold of p = .005 was used.*

|  | *M0 vs M1* | |  | | *M1 vs M2* | | |
| --- | --- | --- | --- | --- | --- | --- | --- |
|  | *χ^2^* | *P* | |  | | *χ^2^* | *P* |
| Anterior thalamic radiation |  |  | |  | |  |  |
| Variance | 0.486 | .784 | |  | | 3.408 | .065 |
| Skewness | **38.864** | **< .0001*** | |  | | 2.087 | .149 |
| Dorsal cingulum |  |  | |  | |  |  |
| Variance | 7.092 | .029 | |  | | 6.506 | .012 |
| Skewness | 2.932 | .231 | |  | | 0.028 | .866 |
| Hippocampal cingulum |  |  | |  | |  |  |
| Variance | 0.923 | .630 | |  | | 0.197 | .657 |
| Skewness | 8.349 | .015 | |  | | 0.066 | .796 |
| Cortiscospinal tract |  |  | |  | |  |  |
| Variance | **24.017** | **<.0001*** | |  | | 0.161 | .688 |
| Skewness | **30.242** | **<.0001*** | |  | | 0.121 | .727 |
| Inferior fronto-occipital fasciculus |  |  | |  | |  |  |
| Variance | 2.085 | .352 | |  | | 0.040 | .841 |
| Skewness | 1.660 | .436 | |  | | 0.814 | .367 |
| Inferior longitudinal fasciculus |  |  | |  | |  |  |
| Variance | 3.613 | .164 | |  | | 6.201 | .013 |
| Skewness | 4.171 | .124 | |  | | 0.871 | .351 |
| Superior longitudinal fasciculus |  |  | |  | |  |  |
| Variance | 5.555 | .062 | |  | | 1.894 | .169 |
| Skewness | 4.692 | .096 | |  | | 3.172 | .075 |
| Uncinate fasciculus |  |  | |  | |  |  |
| Variance | 3.356 | .187 | |  | | 0.001 | .983 |
| Skewness | 1.266 | .531 | |  | | 0.292 | .589 |
| Forceps minor |  |  | |  | |  |  |
| Variance | **16.082** | **.0003*** | |  | | 0.592 | .442 |
| Skewness | 7.721 | 0.021 | |  | | 0.008 | .928 |
| Forceps major |  |  | |  | |  |  |
| Variance | 4.360 | .113 | |  | | 0.537 | 0.463 |
| Skewness | 7.274 | 0.026 | |  | | 0.180 | 0.671 |

**Supplementary Table 2**

*Neuroscience in Psychiatry Network (NSPN) Consortium author list*

| **NSPN Principle Investigators** |
| --- |
| Edward Bullmore  Raymond Dolan  Ian Goodyer  Peter Fonagy  Peter Jones |
| **NSPN (funded) staff:** |
| Michael Moutoussis  Tobias Hauser  Sharon Neufeld  Rafael Romero-Garcia  Michelle St Clair  Petra Vértes  Kirstie Whitaker  Becky Inkster  Gita Prabhu  Cinly Ooi  Umar Toseeb  Barry Widmer  Junaid Bhatti  Laura Villis  Ayesha Alrumaithi  Sarah Birt  Aislinn Bowler  Kalia Cleridou  Hina Dadabhoy  Emma Davies  Ashlyn Firkins  Sian Granville  Elizabeth Harding  Alexandra Hopkins  Daniel Isaacs  Janchai King  Danae Kokorikou  Christina Maurice  Cleo McIntosh  Jessica Memarzia  Harriet Mills  Ciara O’Donnell  Sara Pantaleone  Jenny Scott  Andrea Reiter  Lucy Vanes |
